# Jiangsen Mao: a pioneer in Chinese hepatitis A vaccine and interferon research, a virologist at the forefront of times

**DOI:** 10.1093/procel/pwad043

**Published:** 2023-07-14

**Authors:** Huan Liu, Kunlan Zuo, Zongzhen Wu, Hao Cheng

**Affiliations:** University of Science and Technology of China, Hefei 230026, China; State Key Laboratory of Virology, Wuhan 430072, China; University of Science and Technology of China, Hefei 230026, China; University of Science and Technology of China, Hefei 230026, China; Institute of Microbiology, Chinese Academy of Sciences, Beijing 100101, China

There is a renowned virologist who dedicated his life to virology and medicine research. He has made important contributions to the study of Chinese polio and hepatitis A vaccines, interferons, and the theory of viral reverse transcription. He invented a live attenuated vaccine for hepatitis A (H2 strain) in the late 1970s, which contributed to the prevention of hepatitis A epidemics in China and earned him the title “Hepatitis A Eradicator.” He said, “I was born in a small mountain village and endured many hardships. However, I’ve always tried to alleviate the suffering of people and lived up to the faith in my life.”—Jiangsen Mao (毛江森), a pioneer of hepatitis A vaccine and interferon research in China, a virologist at the Forefront of the Times (Fig. 1).

**Figure 1.**
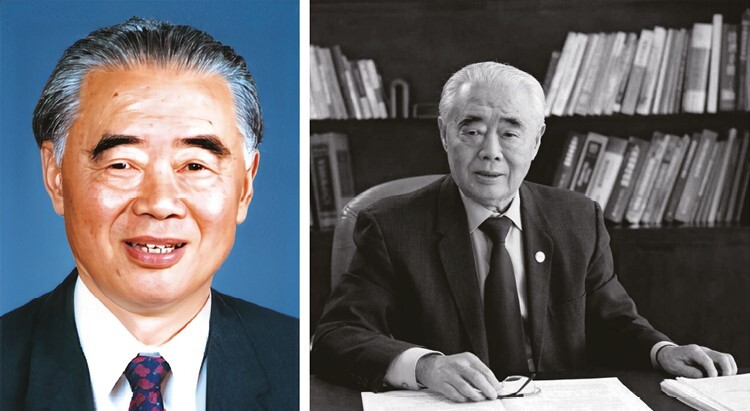
Prof. Jiangsen Mao.

Prof. Jiangsen Mao (1934–2023) was a member of the Chinese Academy of Sciences (CAS). He has received scientific awards for his great contributions to virology, including the Second Prize of the National Invention Award, the First Prize of the Scientific and Technological Progress Award of the Ministry of Health, the title of the National Young and Middle-aged Expert with Outstanding Contribution and the National Advanced Worker.

Prof. Mao was born in Jiangshan County, Zhejiang Province, in 1934. Since childhood, he has suffered from various illnesses due to limited health care and the experiences of his family members with illness. These circumstances influenced his decision to apply to the National Shanghai Medical College (now Shanghai Medical College, Fudan University) in 1951. During his studies, Prof. Mao developed his interests in biochemistry and neurobiology, and since then, he began his career as a medical researcher and virologist.

## Establishment of human embryonic kidney cell line during the study of live attenuated polio vaccine

After graduation in 1956, Prof. Mao was assigned to the Department of Virology at the Chinese Academy of Medical Sciences and participated in the research field of attenuated live polio vaccine-related immunology and virology. From 1957 to 1959, they employed human amniotic membrane cells for the cultivation of poliovirus and observed that the human amniotic membrane underwent digestion without selectivity, often encountering problems such as cell detachment or poor growth. They reported factors affecting the cultivation of human amniotic membrane cells, including variations in quality, low temperature, human serum, and the effect of hydrolyzed casein (Zeng and Mao 1963).

Prof. Mao noted that human embryonic kidney epithelial cells were one of the most sensitive cells to human viruses among known human tissues, but they were not widely used at that time. Since they had not yet obtained a cell strain sensitive to the ECHO virus, Prof. Mao collaborated with Shen He (何申) to establish a primary monolayer cell line derived from human embryonic kidney using trypsin digestion. They collectively established a strain of human embryonic kidney cells (MERN strain). These cells exhibited a higher replication rate and could adapt to regular animal serum and high-pH culture media. They maintained their epithelial cell morphology for 5 months and showed good sensitivity to enteroviruses, which facilitated virus research (Mao et al. 1963). This was the first cell line established by Chinese researchers for virus work, contributing to the study of enteroviruses, measles virus, and hepatitis A virus, and has been continuously passaged and applied to date. They further investigated the effect of heavy water on the replication of LSc-2ab (Sabin attenuated strain) and Mahoney strain in primary and passaged human kidney cells. The study demonstrated that an appropriate concentration of heavy water could increase the viral titer in the cells, which could be applied for the production of live attenuated polio vaccine (Mao and Gu, 1963).

## Pioneer in interferon research

In 1960, Prof. Zhenxiang Huang (黄祯祥), one of the founders of virology in China, nominated Prof. Mao as his assistant. Prof. Mao, aiming to find substances such as antibiotics for the treatment of viral diseases, started his research on interferons. Previous studies have focused on tissue culture systems, with a limited understanding of the conditions for interferon production in intact organisms and its mechanism in protective responses. Prof. Mao and his team conducted studies on interferon production in animals. They selected the viral inhibition substances produced in the Japanese encephalitis virus-mouse system for preliminary observations, which showed minimal production of viral inhibition substances with no significant effects (Mao and Huang, 1963). After 2 years, they discovered that the Japanese encephalitis virus-chick embryo cell system was capable of producing high titers of interferon (Mao et al. 1964).

Prof. Mao speculated that comparing the dynamic production of viruses and interferons during early viral RNA infections or viruses may contribute to understanding the role of RNA in stimulating interferon production. The results indicated that the interferon-inducing factor may be a complex formed by nucleic acids and certain proteins within the viral structure, and viral RNA showed no significant difference in induction, probably due to certain substances in extracts inhibiting interferon production (Mao and Huang 1965a, b). While considering how to increase interferon production, he thought heavy water could enhance the intracellular replication of viruses and might potentially reduce interferon production. He found a decrease in the titer of interferon produced by cells after treatment with heavy water. In addition, viruses propagated in heavy water-treated cells exhibited increased thermal stability, which would have practical implications for enhancing viral titers during infection and preserving viruses (Mao and Huang 1965a, b). They also found that changes in temperature and pH, by affecting interferon production, may be important factors in the mechanism of viral infection recovery (Mao et al. 1966). Given the difficulty of obtaining high concentrations of interferons *in vitro* by using virus and cell systems, he proposed the idea of using biotechnology to improve interferon production.

## “It is possible to transcribe genetic information from RNA to DNA.”

In 1964, Prof. Mao pursued further studies in the Department of Biochemistry at the Institute of Basic Medical Sciences, Chinese Academy of Medical Sciences. While reading a recently arrived journal from the U.S. National Academy of Sciences in the library of Peking Union Medical College, he came across an article by Temin. Temin inoculated a chicken tumor-inducing RNA virus called Rous sarcoma virus (RSV) into Ehrlich ascites tumor cells. In the culture medium after viral infection, in addition to virus-specific RNA, a small amount of virus-specific DNA was also found (Temin 1964). At that time, the “Central Dogma” of molecular genetics stated that genetic information is transcribed from DNA to RNA and then translates into protein. This discovery did not exactly match the “Central Dogma.” He was invited to contribute an article to a journal, and he wrote “Mechanism of Viral Cell Infection,” proposing Temin’s experimental results to a general biological phenomenon. He noted that “it is possible to transcribe genetic information from RNA to DNA,” which made him one of the international scientists for their foresight realizing the significance of reverse transcription, a milestone discovery of virology with far-reaching implications in life sciences.

In 1970, Baltimore and Temin discovered an enzyme in oncogenic RNA viruses that could synthesize DNA using RNA as a template. They named this enzyme RNA-dependent DNA polymerase, also known as reverse transcriptase. The discovery concerning the interaction between tumor viruses and the genetic material of the cell led Baltimore and Temin to be awarded the Nobel Prize in 1975.

## Development of hepatitis A virus vaccine

Hepatitis A virus (HAV) is mainly transmitted via the fecal-oral route, and direct contamination of drinking water and food by HAV in feces can lead to HAV outbreaks or epidemics. In 1978, Prof. Mao was transferred to the Zhejiang Academy of Medical Sciences in Zhejiang Province. At that time, untreated feces were commonly used as fertilizer for crops, providing a direct source of infection for HAV transmission. In Yuanpu village near Hangzhou, 80% of the population was infected, and HAV was prevalent in many rural areas. Due to the lack of valid prevention and treatment, HAV is a serious threat to public health. He made extensive visits and collected samples from patients, from which he isolated the hepatitis A virus. Viral sources and animal models were urgently needed for the study of this disease. Although marmoset monkeys and chimpanzees are susceptible to HAV, they are both precious animals; even more white marmoset monkeys were on the verge of extinction, and China is not the natural habitat of chimpanzees or marmoset monkeys. Subsequently, he and his colleagues discovered that rhesus macaque and stump-tailed macaque were susceptible to hepatitis A virus and caused immune responses, established an animal model for HAV and demonstrated the existence of inapparent infections (Mao et al. 1981).

Since 1978, Prof. Mao and his team have been conducting research on attenuated live vaccines for hepatitis A and using macaques as an animal model to evaluate the virulence of different strains of HAV. They discovered that HAV proliferates in the endoplasmic reticulum of cultured cells, described the morphogenesis of the virus and provided a basis for vaccine development. In the same year, they collected fecal and serum samples from 13 hepatitis A patients in a village near Hangzhou, studying shedding patterns of hepatitis A antigen (HAAg) and antibody response in some cases. The highest percentage of HAAg positivity was in stools collected 1 week before and 1 week after the peak elevation of serum glutamic pyruvic transaminase levels, and peak HAAg shedding in each patient usually occurred in early stools (Mao et al. 1980).

Prof. Mao and his team further investigated the humoral antibody response and early serological diagnosis in patients with hepatitis A. They found that complement-binding antibodies against the hepatitis A virus appeared in serum when serum aminotransferase levels peaked, while immune adherence hemagglutination antibodies began to rise approximately three weeks later. Measurements of serum IgM and IgG levels indicated that the early antibodies were IgM, leading to the establishment of early serological diagnosis using single serum samples for hepatitis A (Mao et al. 1981). His team successfully developed a diagnostic kit for hepatitis A, greatly simplifying the testing procedure, in early 1981. The kit was subsequently utilized in 17 provinces and cities and 40 healthcare units nationwide.

**Figure 2.**
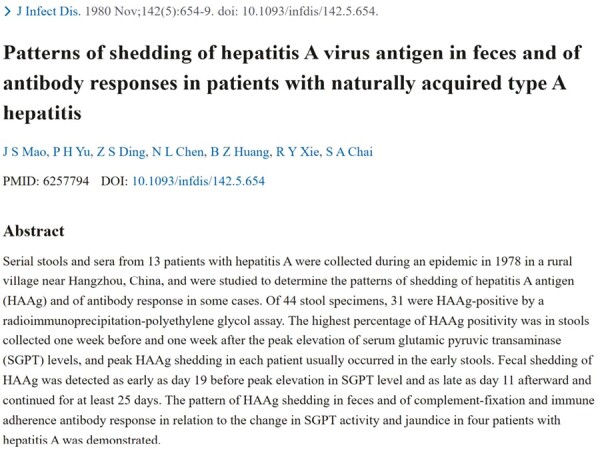
Patterns of shedding of hepatitis A virus antigen in feces and of antibody responses in patients with naturally acquired type A hepatitis.

**Figure 3.**
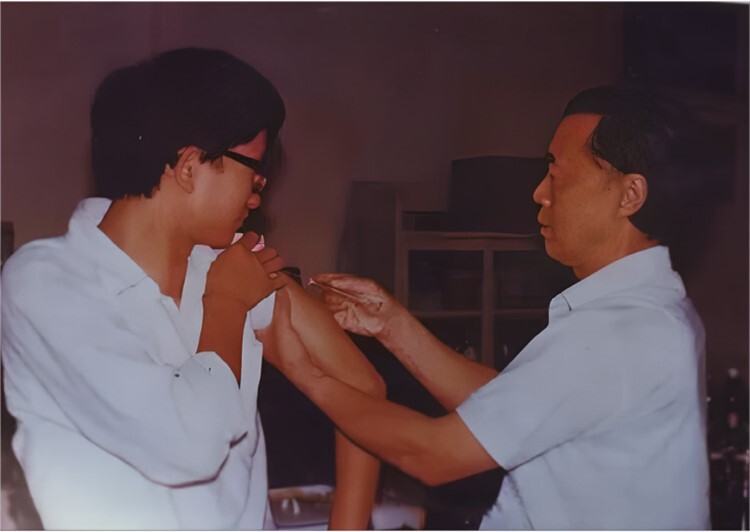
Prof. Jiangsen Mao injected the hepatitis A vaccine for experimental personnel.

HAV vaccine research is needed to select and cultivate effective and safe viral strains. To reduce the virulence of the strain, Prof. Mao conducted low-temperature generation to isolate hepatitis A virus particles in the embryonic kidney cells of red-faced monkeys. After 20 generations of attenuated passages, the strain was further adapted and passaged through seven generations in human embryo lung diploid cells. Finally, a strain suitable for vaccine development, called H2M20K5 (H2), was successfully selected after 2 years. After systematic testing and methodological studies, the results showed that the experimental vaccines produced from this strain were safe and exhibited immunogenicity both in laboratory tests and animal experiments (Fig. 2). Intravenous administration to macaques did not result in abnormal elevation of serum alanine aminotransferase or lactate dehydrogenase isoenzyme 5, and liver biopsies of monkeys showed normal performance. Antibodies in animals began to appear after 6 weeks of inoculation, and all animals produced antibodies in 11 weeks. In May 1987, two phases of clinical vaccination trials were initiated, involving 139 participants, which demonstrated the safety and antibody responses of the attenuated H2 strain (Mao and Dong 1989) (Fig. 3).

In 1992, Prof. Mao proposed the administration of an attenuated live hepatitis A vaccine to combat the epidemic of hepatitis A in China. The live attenuated hepatitis A vaccine (H2 strain) could provide individuals with long-lasting or even lifelong immunity with a single injection. It was quickly put into production in Hangzhou and applied in human inoculation in 1992 (Xin et al. 1998). The use of the attenuated live vaccine against hepatitis A has reached tens of millions of people to date, effectively preventing the occurrence and spread of the disease in China. Sequential studies indicated that the hepatitis A vaccine was one of the live viral vaccines with the fewest side effects. The antibody positive conversion rate reached 93.5%, demonstrating highly immune protective efficacy.

## Moral passion makes me work with great dedication, scientific spirit and method brought me with keys to solve problems

Prof. Jiangsen Mao proposed that scientific judgments were not random guesses but rather based on logical explanations and existing data. He believed in the importance of gathering information, interpreting facts, and building on previous knowledge. During his research on the polio vaccine, he established efficient and sensitive human embryonic kidney cell lines. In his work on interferons, he made discoveries of the mechanism and regulatory functions of antiviral interferons. He had a perspective understanding of scientific principles from the discoveries of life sciences. Successfully developing the hepatitis A vaccine, he contributed to safeguarding the lives and health of people. After studying the clinical and epidemiological characteristics of SARS, he suggested ecological prevention strategies based on the hypothesis that the pathogen originated from wildlife. He once said, “Moral passion makes me work with great dedication, scientific spirit and method brought me with keys to solve problems.” He devoted his life to human health and life science, with moral passion and scientific spirit, and will guide future generations for academic pursuits and value achievements.
